# Electrochemical Storage Behavior of a High-Capacity Mg-Doped P2-Type Na_2/3_Fe_1−y_Mn_y_O_2_ Cathode Material Synthesized by a Sol–Gel Method

**DOI:** 10.3390/gels10010024

**Published:** 2023-12-27

**Authors:** Mobinul Islam, Md. Shahriar Ahmed, Daseul Han, Gazi A. K. M. Rafiqul Bari, Kyung-Wan Nam

**Affiliations:** 1 Department of Energy & Materials Engineering, Dongguk University, Seoul 04620, Republic of Koreaendend42@naver.com (D.H.); 2 School of Mechanical Smart and Industrial Engineering, Gachon University, Seongnam 13120, Republic of Korea; grafiqulbari@gachon.ac.kr

**Keywords:** sol–gel, gel precursor, sodium-ion battery, energy storage, cathode material

## Abstract

Grid-scale energy storage applications can benefit from rechargeable sodium-ion batteries. As a potential material for making non-cobalt, nickel-free, cost-effective cathodes, earth-abundant Na_2/3_Fe_1/2_Mn_1/2_O_2_ is of particular interest. However, Mn^3+^ ions are particularly susceptible to the Jahn–Teller effect, which can lead to an unstable structure and continuous capacity degradation. Modifying the crystal structure by aliovalent doping is considered an effective strategy to alleviate the Jahn–Teller effect. Using a sol–gel synthesis route followed by heat treatment, we succeeded in preparing an Mg-doped Na_2/3_Fe_1−y_Mn_y_O_2_ cathode. Its electrochemical properties and charge compensation mechanism were then studied using synchrotron-based X-ray absorption spectroscopy and in situ X-ray diffraction techniques. The results revealed that Mg doping reduced the number of Mn^3+^ Jahn–Teller centers and alleviated high voltage phase transition. However, Mg doping was unable to suppress the P2-P’2 phase transition at a low voltage discharge. An initial discharge capacity of about 196 mAh g^−1^ was obtained at a current density of 20 mAh g^−1^, and 60% of rate capability was maintained at a current density of 200 mAh g^−1^ in a voltage range of 1.5–4.3 V. This study will greatly contribute to the ongoing search for advanced and efficient cathodes from earth-abundant elements for rechargeable sodium-ion batteries operable at room temperature.

## 1. Introduction

Population growth, technological advancement, and industrialization have led to a substantial increase in energy demand. Fossil fuels have been the primary energy source for the past few decades. However, alternative energy sources that could replace fossil fuels are needed to preserve our planet and achieve green energy for sustainable human development. There is an increasing interest in renewable energy sources such as wind, solar, etc. However, such renewable energy sources have inherent limitations related to their intermittency and geographic dispersion that need large-scale electric energy (grid storage) systems. Batteries are considered the most promising option for grid storage in the future due to their low operating costs and long life cycles [1]. Lithium-ion batteries (LIBs) have the highest energy density among modern batteries. However, lithium is a scarce element in the Earth’s crust, and it is not widely distributed [2]. Additionally, the use of portable electronics and electric vehicles has increased exponentially in recent years, resulting in an increased demand for lithium supplies and price hikes. Alternatively, sodium-ion batteries (SIBs) could complement LIBs due to abundant available sources and suitable redox potential of sodium ions (E_(Na/Na+)_ = −2.71 V vs. SHE) [3]. SIBs are ideal grid-scale storage solutions where battery energy density is not a major factor. However, the lack of appropriate and cost-effective cathode materials that can provide high capacity and excellent cycle stability has made it difficult to utilize SIBs in practical applications.

Various types of SIB cathode materials have been reported, with layered-type oxide materials (NaxTMO_2_ = Mn, Co, Fe, Ni, Ti, Cu, Cr, or mixtures of these elements) being the most promising due to their large capacities and tunable properties [4]. Layered oxides with Fe and Mn as redox centers in positive electrode materials have special importance because both Fe and Mn are cheap, abundant, and environmentally friendly. It is important to note that although Na is ten times cheaper than Li, the per-molar cost of transition metals Co is four times higher, and Ni is 0.7 times higher than that of Li. Therefore, the economic competitiveness of SIBs will be greatly diminished if they use a significant fraction of Co and Ni. According to the nomenclature proposed by Delmas et al. [5], layered oxides are categorized into two main groups: O3 and P2. The P2-Na_x_FeO_2_ phase does not exist. Although O3-type NaFeO_2_ is electrochemically active, its reversible capacity (90 mAh g^−1^) is less than half of its theoretical capacity (242 mAh g^−1^) [6]. P2-Na_0.67_MnO_2_ provides a substantial initial capacity of 170 mAh g^−1^, which is close to its theoretical capacity [7]. Despite this, Na_x_MnO_2_ is subject to an irreversible P2→OP4 phase transition due to the gliding of MnO_2_-slabs, which can lead to a decay of capacity with time [8]. Compared to a P2 phase, an O3-NaMnO_2_ phase contains more sodium with a greater capacity. However, O3-type oxides are easily contaminated due to hygroscopic degradation. They are also prone to more complex phase transitions during the electrochemical process, which can cause unsatisfactory cycling performance [9]. A P2-type oxide exhibits relatively high resistance to hygroscopic degradation. Thus, it can maintain its original structure over a wide voltage range. In other words, P2-type oxides have a significant practical advantage in handling and processing.

A combination of Na_x_FeO_2_ and Na_x_MnO_2_ is also an attractive choice as a cobalt-free cathode. It is noteworthy that the P2-type Na_2/3_Fe_1/2_Mn_1/2_O_2_ has a relatively high initial capacity of 190 mAh g^−1^, higher than all Fe or Mn-based layered oxides reported [6,7,8,9,10]. Several other derivatives of Fe/Mn-based combined layered oxides have also been investigated since the first report of auspicious properties of P2-Na_2/3_Fe_1/2_Mn_1/2_O_2_ [10]. Na_2/3_Fe_2/3_Mn_1/3_O_2_, a Fe-rich derivative with a P2-type structure, has an initial reversible capacity of approximately 151 mAhg^−1^ [11]. In Na_2/3_Fe_2/3_Mn_1/3_O_2_, Mn is used as both a promoter and a stabilizer for P2-phase reactions because it is impossible to synthesize P2-Na_2/3_FeO_2_. Mn content is likely to be associated with better electrochemical performance. For example, a Mn-rich layered system P2-Na_2/3_Fe_1/3_Mn_2/3_O_2_ can deliver a reversible capacity of 193 mAhg^−1^ [12]. Nevertheless, P2-type Na_x_Fe_1−y_Mn_y_O_2_ cathode systems suffer from poor capacity retention, hindering their practical and commercial applications. Research has identified irreversible phase changes as a major contributing factor to its poor capacity retention [13,14]. An SIB cathode material is usually designed with the goal of either increasing energy density or seeking structural stability. By doping/substituting some foreign elements, such as Al, Ca, Ti, Cr, and Mg, the crystal structure can be stabilized, and phase transformations can be restrained in the case of P2-type layered oxide materials, resulting in a more stable cycle [15,16].

The majority of Na-Fe-Mn-O-based cathode samples have been synthesized by conventional solid-state methods [17]. Sol–gel synthesis offers more control over synthesis parameters than solid-state synthesis. It is possible to fine-tune sol–gel routes by assessing many independent synthesis parameters (e.g., gelling temperature, gel aging, chelating agent, gel formation solution composition, gel precursor nature, precursor ratio, solution environment, and annealing conditions) to prepare specific crystal morphologies and defects [18]. In the present study, we present a citric-assisted sol–gel synthesis of P2-Na_2/3_Mg_2/9_Fe_2/9_Mn_5/9_O_2_ (NFM-Mg), an Mg-doped Mn-rich earth-abundant cathode with a P2 structure, and its ability to reversibly work in sodium-ion batteries using an aprotic electrolyte. Different studies are in progress to decouple the effect of various chelating agents and the nature of the gel precursors (chelate gel and organic polymeric gel) on particle morphology and charge storage capability of the studied material. The results will be published elsewhere. A P2-type Na_2/3_Fe_1/2_Mn_1/2_O_2_ (NFM) cathodes contain both Mn^3+^ and Mn^4+^ ions. Mn has an average oxidation state of 3.66. When Mg doping is applied to the NFM-Mg cathode, the Mn oxidation state is pushed toward 4+, which minimizes the number of Mn^3+^ Jahn–Teller centers and delays or erases the high voltage phase transition that is typically experienced on P2-Na_2/3_Fe_1/2_Mn_1/2_O_2_ and P2-Na_2/3_MnO_2_ cathodes [8,13]. The major goal of this manuscript is to shed light on the NFM-Mg charge compensation mechanism to understand and search for ways to enhance structural stability and minimize capacity fading in a Na-Fe-Mn-O-based cathode system upon cycling.

## 2. Results and Discussion

### 2.1. Crystal Structure

**Figure 1:**
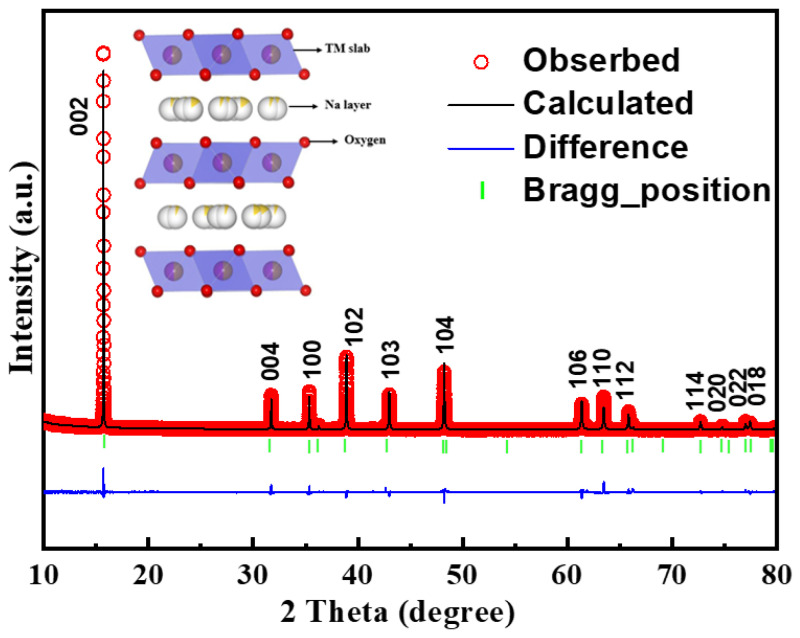
Rietveld refinement result for the P2-Na_2/3_Mg_2/9_Fe_2/9_Mn_5/9_O_2_ compound on the synchrotron high-resolution powder diffraction (HRPD) data. The brag positions correspond to the hexagonal P2-Na_2/3_Fe_1/2_Mn_1/2_O_2_ phase (P6_3_/mmc).

**Scheme 1:**
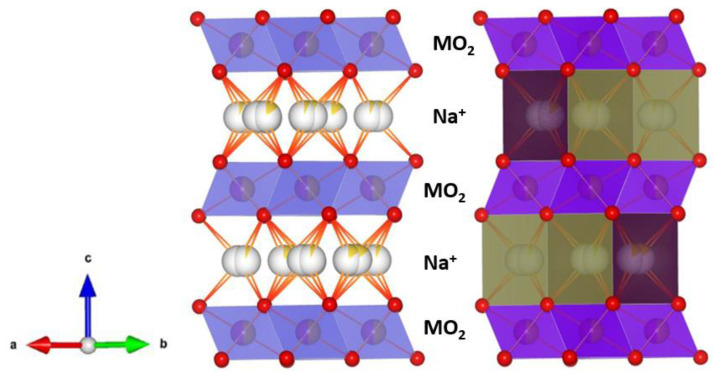
Schematic illustration of P2-Na_2/3_Mg_2/9_Fe_2/9_Mn_5/9_O_2_ structure.

The Rietveld refinement on the synchrotron X-ray diffraction (XRD, Rigaku, Tokyo, Japan) data of the prepared sample is presented in Figure 1. The XRD pattern can be fully indexed to the hexagonal P6_3_/mmc space group, with diffraction peaks mainly at 2θ = 15.63°, 31.67°, 35.42°, 38.86°, 43.01°, 48.30°, 61.37°, 63.48°, and 65.88°. The refinement is in good agreement with the P2-single phase [10], and all the peaks are identical to the standard values of Na_2/3_Fe_1/2_Mn_1/2_O_2_ (ICSD No. 194-731). There was no noticeable impurity or broadened peaks evident across the entire structure. Prominent and sharp diffraction lines indicated that the as-synthesized compound was highly crystalline. The refined lattice parameters were *a* = 2.91062 Å and *c* = 11.19962 Å, as shown in Table 1. The reliability R-factors were R_wp_ = 15.4 and R_exp_ = 7.62; nevertheless, this is not a highly ideal result, which may be due to the uncontrollable stacking faults in layered compounds. The crystal structure of the prepared sample consisting of ordered stacked MO_2_ (M = Fe, Mn, and Mg) and Na layers is shown in Scheme 1. It could be defined as prismatic 2 (P-2) because Na^+^ ions occupied two different sites (Na_f_ and Na_e_) between prismatic AB-BA-type layers.

**Figure 2:**
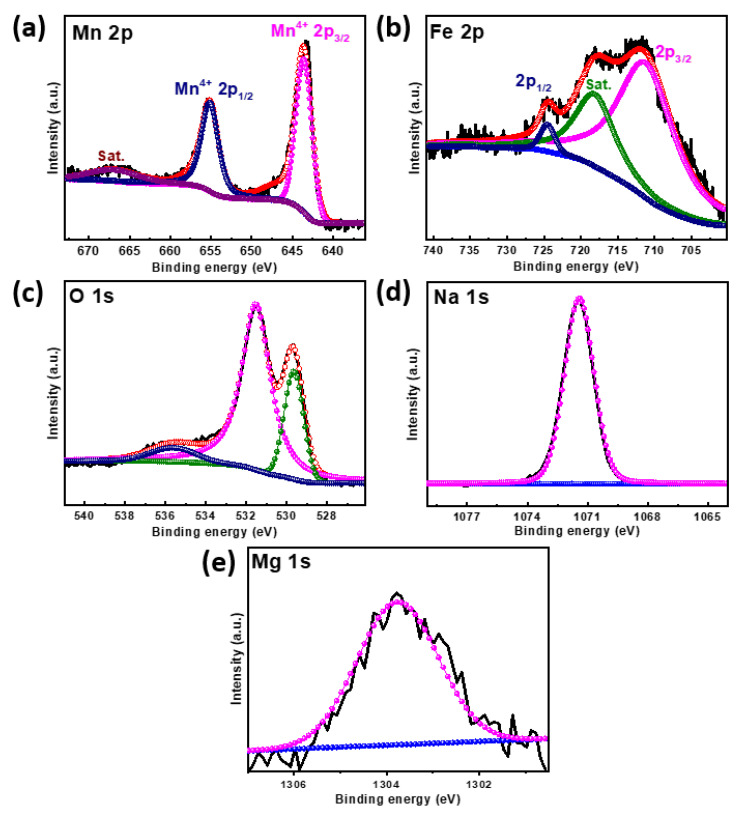
XPS analyses of P2-Na_2/3_Mg_2/9_Fe_2/9_Mn_5/9_O_2_ material: the high-resolution XPS spectra of (**a**) Mn 2p, (**b**) Fe 2p, (**c**) O 1s, (**d**) Na 1s, and (**e**) Mg 1s.

Surface-sensitive X-ray photoelectron spectroscopy (XPS) was applied to clarify the valence states and composition of the as-synthesized P2-Na_2/3_Mg_2/9_Fe_2/9_Mn_5/9_O_2_ material, and the results are shown in Figure 2. The deconvoluted XPS spectra of Mn 2p (Figure 2a) are fitted into two peaks at 643.67 eV and 655.1 eV, which can be assigned to Mn^4+^ (2p_3/2_) and Mn^4+^ (2p_1/2_) species, respectively [19,20]. The Fe 2p spectrum exhibits two peaks at 724.4 and 711.1 eV, corresponding to Fe 2p_1/2_ and Fe 2p_3/2_, respectively (Figure 2b). These peaks can be indexed to the Fe (+3) oxidation state [21]. The O 1s spectrum can be fitted into three components (Figure 2c), which can be attributed to the lattice oxygen (529.6 eV) and to surface-adsorbed species (531.5 and 535.6 eV) [20]. Figure 2d,e show the XPS spectra of the Na 1s and Mg 2p peaks, respectively, with binding energies of 1071.5 and 1303.75 eV, confirming the presence of Na^+^ and Mg^2+^ ions in the as-synthesized P2-Na_2/3_Mg_2/9_Fe_2/9_Mn_5/9_O_2_ material.

**Figure 3:**
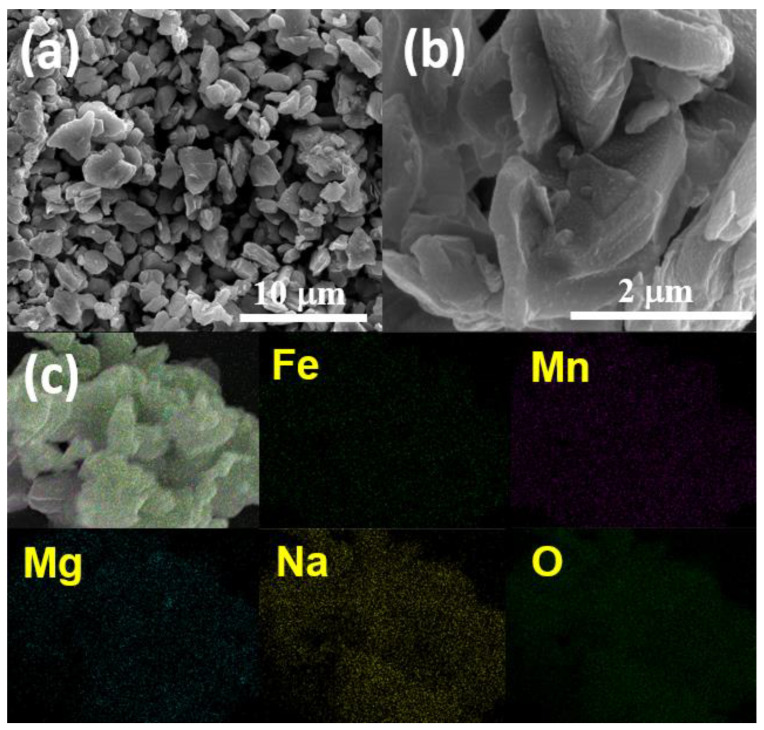
SEM micrographs of as-synthesized P2-Na_2/3_Mg_2/9_Fe_2/9_Mn_5/9_O_2_ at (**a**) low and (**b**) high magnification and (**c**) corresponding SEM-EDS elemental mapping images.

Morphology of the P2-type Na_2/3_Mg_2/9_Fe_2/9_Mn_5/9_O_2_ sample synthesized at 850 °C was examined by SEM. The SEM image is shown in Figure 3. Particles in the sample exhibited a uniform distribution. They were mainly plate-shaped sub-micron particles with sizes ranging from 1 μm to 4 μm, often seen for a P2-layered structure [16,22,23]. The atomic compositions of the sample were measured by plasma-optical emission spectroscopy (ICP-OES), and the results are listed in Table 2. The as-prepared sample has an atomic ratio that is almost identical to the designed stoichiometric ratio. In addition, we utilized energy-dispersive X-ray spectroscopy (EDS) coupled with SEM to check the elemental distribution and atomic composition of the as-prepared sample. Figure 3c shows that all elements, such as Mn, Fe, Mg, Na, and O, are homogeneously distributed within the sample. Table S1 shows that the atomic ratio of Mg, Fe, and Mn is 0.22:0.21:0.55. These values are very close to the projected values of these elements in the P2-Na_2/3_Mg_2/9_Fe_2/9_Mn_5/9_O_2_ material.

### 2.2. Electrochemical Performance

**Figure 4:**
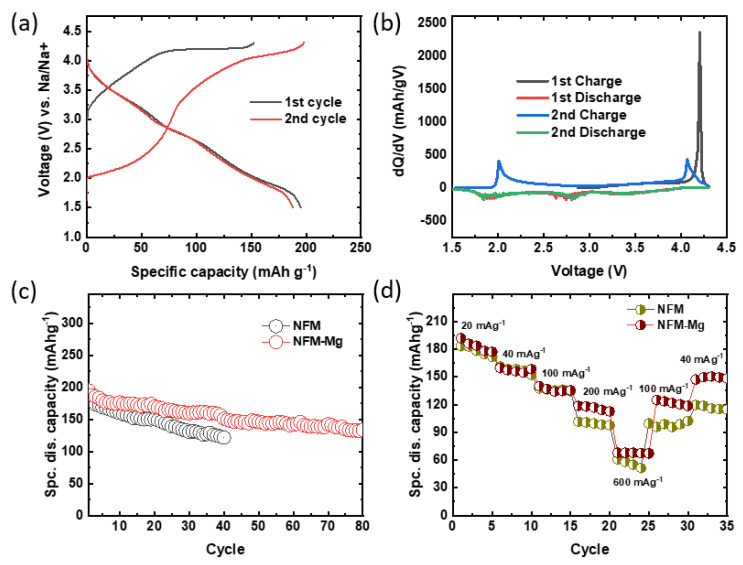
(**a**) Galvanostatic voltage profiles; (**b**) dQ/dV curves of the NFM-Mg cathode material; (**c**) cycling; (**d**) rate capability performance comparison between P2-Na_2/3_Fe_1/2_Mn_1/2_O_2_ (NFM) and P2-Na_2/3_Mg_2/9_Fe_2/9_Mn_5/9_O_2_ (NFM-Mg) cathode materials in Na-ion half-cell.

Charge valance of the benchmark compound, P2-Na_2/3_Fe^3+^_1/2_Mn^3+^_x_Mn^4+^_1/2−x_O_2_ (NFM), indicates the presence of Jahn–Teller active Mn^3+^ ions. Our designed composition formula of P2-Na_2/3_Mg^2+^_2/9_Fe^3+^_2/9_Mn^4+^_5/9_O_2_ (NFM-Mg) was supposed to consist of non-Jahn–Teller Fe^3+^ and Mn^4+^ ions. Electrochemical performance of the designed NFM-Mg as a positive electrode material for Na-ion batteries was evaluated against Na metal anodes with a liquid electrolyte of 1.2 M NaPF_6_ in 9:9:2 *v*/*v* ethylene carbonate/dimethyl carbonate (DMC)/propylene carbonate (PC) of coin cells. Figure 4a shows typical charge and discharge profiles of the NFM-Mg cathode at 20 mA g^−1^. The NFM-Mg electrode voltage curve exhibited a slope accompanied by a well-defined voltage plateau at 4.2 V, which could be attributed to Fe^3+^/Fe^4+^ and/or oxygen redox reactions during the first charge. Considering that NFM-Mg has tetravalent Mn to maintain charge balance, its oxidation from Mn^4+^ to Mn^5+^ would be impossible because Mn^5+^ is not stable in octahedron coordination. The NFM-Mg voltage curve during the first charge was quite different from that of NFM (Figure S2a). The NFM had a two-step slope corresponding to Mn^3+^/Mn^4+^ and Fe^3+^/Fe^4+^ [10,13]. The appearance of the voltage plateau (at ca. 4.2 V vs. Na) in Figure 4a reminds us about anionic redox (O^2−^/O^n−^) based Na cathode material’s electrochemical behavior in Na-cell [24,25]. It suggests that oxide ions might contribute to the redox process. The NFM-Mg delivered ≈152.5 mAh g^−1^ of initial charge capacity and ≈196 mAh g^−1^ of discharge capacity between 1.5 V and 4.3 V. Note that the theoretical capacity based on a single-electron redox process of a Fe^3+^/Fe^4+^ redox couple is only ≈45 mAh g^−1^. This means that the capacity during the initial charge process might be attributed to oxygen redox (O^2−^/O^n−^). The second charge and discharge capacities of the NFM-Mg cathode were 198.5 and 190 mAh g^−1^, respectively. On the other hand, the NFM delivered ≈112.5 mAh g^−1^ of initial charge capacity and ≈183 mAh g^−1^ of discharge capacity, followed by the second charge (≈180.7 mAh g^−1^) and discharge (≈178.3 mAh g^−1^) capacities at the same condition (Figure S2a). In comparison with the NFM performance, the NFM-Mg clearly showed a better charge storage capacity. Anodic and cathodic peaks and their evolution upon cycling were more visible in the dQ/dV plots, as shown in Figure 4b and Figure S2b. The high voltage region showed one anodic peak at around 4.2 V during the first charge, which shifted to 4.05 V in the following charge cycle, consistent with the voltage profile shown in Figure 3a. A broad cathodic peak at around 2.8 V in the differential capacity curve corresponded to oxygen reduction [26]. It was about 1.5 V away from the oxidation peak. The reasons for this strong voltage hysteresis are unknown at this point. In contrast, the Fe^3+^/Fe^4+^ redox peaks at around 4.1 and 3.25 V dominated the high-voltage region of the NFM cathode material (Figure S2b). The redox peaks in the low-voltage region of the NFM electrode are associated with the Mn^3+^/Mn^4+^ redox reaction. Although there were no redox peaks associated with the Fe^3+^/Fe^4+^ redox reaction [23], a pair of anodic/cathodic peaks at around 2.0 and 1.85 V associated with the Mn^3+^/Mn^4+^ redox reaction dominated the low-voltage region of the NFM-Mg cathode. A detailed charge compensation mechanism is discussed in the next section. The reversible capacity gradually dropped from the initial 196 mAh g^−1^ to 162 mAh g^−1^ at the 30th cycle and 134 mAh g^−1^ at the 80th cycle, as shown in Figure 4c. After 40 cycles, the Mg-doped NFM electrode exhibited better capacity retention (≈77%) than the undoped NFM electrode (≈66%), and the 82% capacity retention of the NFM-Mg electrode after 30 cycles at 20 mA g^−1^ was marginally better than that described in the study of Yabuuchi et al. [10]. They reported that approximately 80% capacity retention was obtained at a rate of 12 mA g^−1^ (after 30 cycles) under the same voltage range. The rate capability of the NFM and NFM-Mg cathodes is shown in Figure 4d. The NFM-Mg cathode material could release average reversible discharge capacities of 158, 137, 117, and 68 mAh g^−1^ at 40, 100, 200, and 600 mA g^−1^, respectively. It is evident that the Mg-doped NFM electrode demonstrates improved rate capability compared with the NFM electrode, particularly at high current densities. The NFM-Mg electrode could retain approximately 60% of its reversible capacity when the current density was increased ten times (from 20 to 200 mAh g^−1^). This rate performance is impressive for a layered Fe/Mn-based Na-ion battery cathode. The interlayer spacing of the Mg-doped sample usually becomes larger due to a larger radius of Mg (2+) ions than those of Mn and Fe cations, which might lead to a faster diffusion of Na^+^ ions [27].

### 2.3. Charge Compensation and Crystal Structure Evolution

We measured NFM-Mg electrode samples with various electrochemical states during the first one-and-a-half cycles using synchrotron ex situ spectroscopic techniques to understand better the roles played by Fe, Mn, and O in electrochemical processes and comprehend the charge compensation mechanism. Hard X-ray absorption spectroscopy (hXAS) spectra were collected on Mn and Fe K-edges to examine variations in valence state and evolution of local structure. To examine how the electronic structure of the TM3d-O2p hybrid changed under different states of sodium intercalation/deintercalation, ex situ soft X-ray absorption spectroscopy (sXAS) spectra were also collected on Mn L_2,3_-edge and O K-edge. Herein, TM was defined as transitional metals (Mn, Fe).

**Figure 5:**
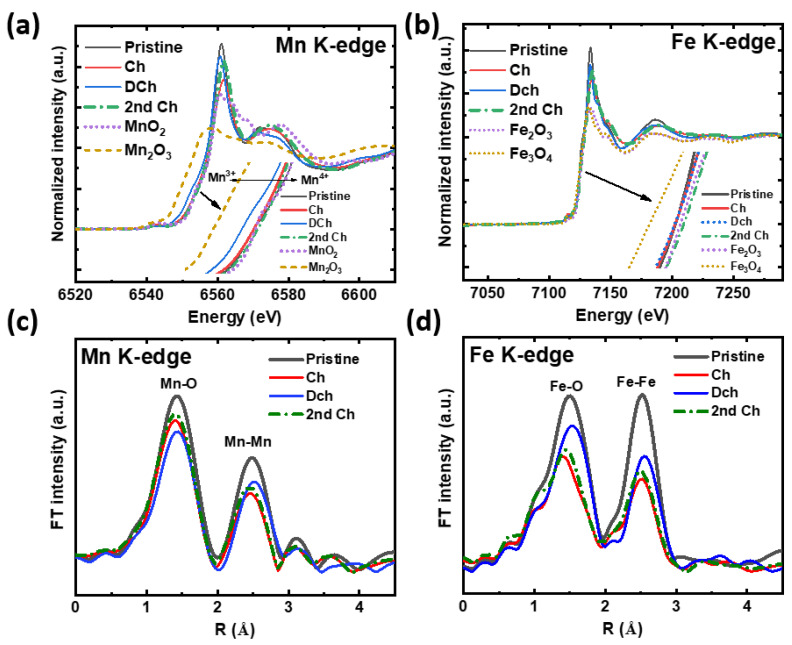
Ex situ XAS analysis of NFM-Mg electrodes at different state of charges during the first and half electrochemical cycle: (**a**) XANES and (**b**) corresponding EXAFS spectra at Mn K-edge; (**c**) XANES and (**d**) corresponding EXAFS spectra at Fe K-edge. Electrodes collected at the end of first charge, discharge, and second charge.

Figure 5a,b present normalized X-ray absorption near edge structure (XANES) spectra of Mn and Fe K-edges, respectively, at different sodiation and desodiation states. The valence state of Fe in the pristine sample was approximately 3+ based on a comparison between Fe K-edge XAS spectra of the pristine sample and standard reference compounds. On the other hand, Mn in NFM-Mg had a valence close to 4+ in comparison with the standard reference spectrum. During initial charging from OCV (pristine) to 4.3 V (Full Ch), the Mn K-edge showed a very small change in absorption peak shape, which could not be attributed to oxidation change. It was surprising that the Fe K edge did not shift towards higher energy regions during initial charging, indicating that the Fe valence state remained unchanged (≈+3). This suggested that the initial charge capacity came from only a redox reaction on oxygen anions, as discussed in the previous section. It was found that the Fe K-edge again remained static upon discharge. On the other hand, Mn K-edge substantially shifted to lower energies after discharge to 1.5 V, indicating that Mn was reduced to a lower valence state at the end of the discharge process. It appeared that the first discharge capacity mainly resulted from the reduction in Mn ions. After the second charge, the spectral position of the Mn K-edge was almost identical to that of MnO_2_, indicating that the Mn^4+^ oxidation state was reestablished. In the second charge, the Fe K-edge moved to a slightly higher energy position, suggesting that Fe had a very minimal contribution to the second charge capacity. Studies of those redox behaviors during long-range cycling are in progress. Figure 5c,d show the evolution of Fourier-transformed extended X-ray absorption fine structure (FT-EXAFS) spectra with cycling. Both Fe and Mn K-edge EXAFS showed two strong peaks. The first peak between 1 and 2 Å was related to the average TM-O length of the first shell TM-O6 octahedron. The second peak between 2 and 3 Å was related to bonds between transition metals in the second shell of the TM-TM6 hexagon. From initial charge (4.3 V) to full discharge (1.5 V) to second charge up to 4.3 V, all interatomic Mn-O, Fe-O, and TM-TM bond lengths underwent a reversible decrease/increase upon desodiation/sodiation. A considerable displacement of these interatomic bond distances from the pristine sample indicated a distortion of Mn and Fe atoms’ local environment during the insertion/extraction of Na^+^ ions, possibly induced by irreversible oxygen loss.

**Figure 6:**
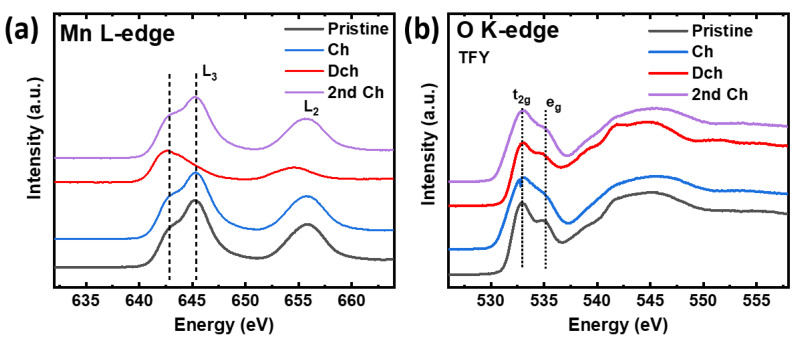
NFM-Mg cycled electrodes: (**a**) Mn L_2,3_-edge XAS in TEY mode; (**b**) O K-edge XAS in TFY mode.

Metal L_2,3_-edge absorption peaks resulting from electric dipole-allowed 2p→3d transition are sensitive to the oxidation state and spin state [28]. Total electron yield (TEY) mode was used to collect sXAS data at the Mn L_2,3_-edge, as shown in Figure 6a. Splitting of the high energy range (metal L_2_-edge) and low energy range (metal L_3_-edge) corresponded to the interaction and respective electronic transitions from Mn 2p_1/2_ and 2p_3/2_ core levels to highly hybridized O2p-Mn3d orbitals, respectively. The Mn L_2,3_ spectral feature of the pristine NFM-Mg sample resembled that of MnO_2_ [29], suggesting that Mn was in its tetravalent (4+) state. The Mn L-edge sXAS did not change significantly after charging the NFM-Mg electrode to 4.3 V, indicating that Mn ions were maintained in their Mn 4+ states after desodiation. After sodiation to 1.5 V, the peak of Mn L_3_-edge significantly shifted to a lower energy area, suggesting that the metal was being reduced from 4+ to a lower valence. The spectral shape of Mn L_2,3_-edge after the second charge was identical to that of the first charge, which again evidenced the reversible oxidation/reduction in the Mn^3+^/Mn^4+^ redox couple.

Next, O K-edge sXAS data were collected in bulk-sensitive total fluorescence yield (TFY) mode. Results are presented in Figure 6b, where two distinct regions can be distinguished from the spectra. A pre-edge feature below 535 eV corresponded to an excitation from core-level O 1s orbitals to empty and hybridized (with Metal 3d) O 2p orbitals. The broad and wide hump above 535 eV represented the transition from O 1s orbitals to O 2p-TM 4sp hybridized states. Due to this, an O K-edge sXAS feature cannot be used directly to prove oxygen redox reactions. Instead, mapping of resonant inelastic X-ray scattering (mRIXS) is a powerful tool for exploring the lattice oxygen redox because this technique disentangles the intrinsic oxygen redox from the TM-O hybridization [30,31,32]. The sXAS O K-edge was only a measure of the change in TM-O hybridization strength. Valence states of TM dominated spectral line shapes of two pre-edge peaks assigned to t_2g_ and e_g_ states [26]. In Figure 6b, the dip between t_2g_ and e_g_ states were filled during the first charge process. Note that the lattice oxygen redox could be confirmed by identifying the emergence of a specific feature between t_2g_ and e_g_ peaks in the excitation energy coordinate system in mRIXS [32]. This filled dip might indirectly indicate that lattice oxygen redox participated in charge compensation during the first charge process. Because of electron filling in the t_2g_ energy, the intensity ratio of t_2g_ and e_g_ states decreased after discharge. During the second charge, the intensity ratio increased again, but the dip between t_2g_ and e_g_ states remained unfilled. The exact explanation for this phenomenon is ambiguous at this stage. Nevertheless, in combination with the electrochemical (dQ/dV) analysis discussed earlier, it was evident that there was a substantial difference between the first cycle and the second cycle’s lattice oxygen contributions to the charge compensation process.

**Figure 7:**
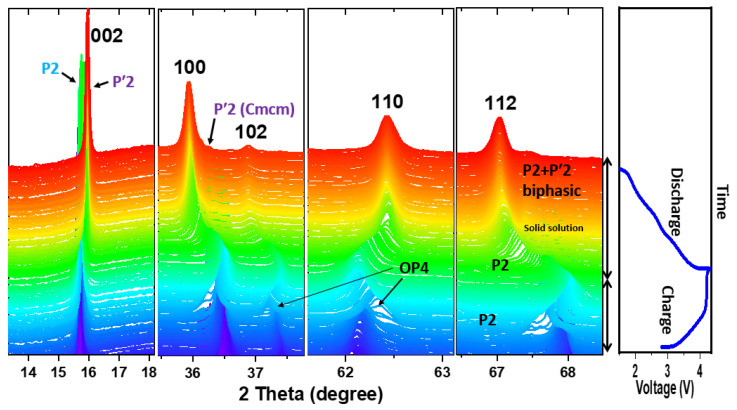
In situ synchrotron XRD of NFM-Mg electrode during the first charge/discharge process at a current density of 10 mA g^−1^. The electrode charged to 4.3 V (P2 phase, blue color) and then discharged to 1.5 V. Discharge at the initial stage continues by maintaining the P2 phase (green color). Eventually, a P2 + P′2 biphasic region (red color) with an intermediate solid solution (yellow color) region appears.

To study structural rearrangements caused by cycling in the material, a synchrotron-based in situ XRD experiment was carried out. The experiment started with the material in the OCV state (a fresh electrode), which corresponds to the composition P2-Na_2/3_Mg_2/9_Fe_2/9_Mn_5/9_O_2_. The results are shown in Figure 7. The P2-type hexagonal structure of the NFM-Mg cathode material was maintained during electrochemical desodiation. During the timespan of charge (desodiation), P2 (100), (102), and (112) peaks gradually changed directions towards higher angles, while P2 (002) and (110) peaks deviated towards lower angles. Moving peaks were correlated with decreasing a-axis and increasing **c**-axis. As oxygen anions are repelled between two transition-metal layers during Na^+^ extraction (charge), the interlayer distance between them increases, which corresponds to **c**-axis expansion. Notably, when the cell was discharged, parameters returned in the original direction, indicating that the structure was reversible. The P2 phase (blue color) is retained throughout the whole charge process, except for a stacking fault (OP4 phase) formation. The origin of this intermediate OP4 phase can be attributed to irreversible oxygen loss. However, the detrimental “Z/OP4 phase”, which is usually observed at around 2θ ≈ 17° for the P2-Na_2/3_Fe_1−y_Mn_y_O_2_-type compound [13,33] when cycling above 4.0 V vs. Na^+^/Na, did not appear here. A P2-to-“Z”/OP4-type phase transition associated with large volume changes is accountable for capacity fading [34]. In short, the introduction of Mg^2+^ ions into the crystal structure successfully suppressed unknown “Z” phase formation. It appears that the P2 phase (green color) continues at the initial stage of the discharge process. Nevertheless, discharging toward a lower voltage of ~1.5 V, an additional phase (orthorhombic P′2, space group: Cmcm) gradually appeared, accompanied by a reduction in Mn^4+^ ions, which was evidenced by a splitting of (002) and (100) peaks. Figure 7 clearly identifies the biphasic mechanism (red and green mixed colors) of the discharge process, which occurs after an intermediate solid solution (yellow color) region. It has been reported that P2 and P’2 phases have a significant volume difference (4.96 Å^3^) in the low voltage (~1.5 V) region [35], which could cause serious structural separation. It might also lead to mechanical strain during cycles. In summary, our in situ XRD results illustrate that Mg-doping in a P2 type Na_2/3_Fe_1−y_Mn_y_O_2_ compound can significantly inhibit unknown “Z” phase formation during desodiation without suppressing P2-P’2 phase transition during sodiation at low voltages.

Overall, the results of the above experiments indicate that oxygen is the only element involved in the initial charging process, while oxygen and manganese are both involved in the subsequent discharge process. Mg doping remediated the Jahn–Teller active Mn^3+^ ions in a P2-Na_x_Fe_1−y_Mn_y_O_2_ system that caused phase transition during high voltage desodiation associated with gliding of MnO_2_-slabs and decaying capacities. It was certain that the probability of Jahn–Teller active Fe^4+^ ions forming at high voltage regions was reduced because Fe^3+^ ions could not be oxidized during desodiation here. However, irreversible oxygen activity is an issue that should be resolved in future studies. The 68% capacity retention after 80 cycles during electrochemical cycling was satisfactory compared to several previous reports [11,17,23]. Nevertheless, this material’s performance could be further improved through simultaneous doping and coating approaches.

## 3. Conclusions

In this study, we applied a sol–gel method for synthesizing a new cathode material, P2-Na_2/3_Mg_2/9_Fe_2/9_Mn_5/9_O_2_. We then tested its electrochemical performance in Na half-cells. This material showed a high initial reversible capacity of 196 mAh g^−1^ and maintained a reversible capacity of mAh g^−1^ after 80 cycles with a capacity retention of 68% within a wide potential range of 1.5V to 4.3 V. Additionally, it had good rate capabilities, providing 117 mAh g^−1^ of reversible capacity at a current density of 200 mA g^−1^. Synchrotron XAS results confirmed that its capacity was due to single cationic (Mn^3+^/Mn^4+^) redox in addition to partially reversible anionic redox (O^2−^/O^n−^) in short-term cycling. Desodiation at high voltage resulted in no undesired phase transition and structural distortion, as evidenced by in situ XRD analysis because of the presence of Jahn–Teller inactive Fe^3+^ (t^3^_2g_e^2^_g_) and Mn^4+^ (t^3^_2g_ e^0^_g_) ions. Irreversible oxygen loss can be minimized by altering dopant concentration, which is left to be resolved in future work. In terms of developing SIBs’ layered cathode materials, these results are quite valuable.

## 4. Materials and Methods

The NFM-Mg was prepared using the sol–gel method using corresponding metal nitrate salts as source materials. All chemicals used in this study, including NaNO_3_ (99%), Fe(NO_3_)_3_∙9H_2_O (99%), Mn(NO_3_)_2_∙4H_2_O (99%), Mg(NO_3_)_2_∙6H_2_O, and citric acid, were purchased from Junsei Chemical Co. Ltd., Japan. We first solubilized nitrate salts in distilled water and then added them to a citric acid solution at room temperature at a molar ratio of 0.5:1 (citric acid:cations). The mixture was then stirred continuously for 1 h. The prepared mixture was then placed on a hot plate at a temperature of 80 °C. The solution was mixed continuously by magnetic stirring for a certain period and converted to gel, which involved a simple reaction of hydrolysis and condensation. The resultant gel was quickly transferred and placed overnight in a vacuum oven at 120 °C. For auto-combustion of citric acid, the gel was further heated at 200 °C. In a box furnace, resulting powders were pre-heated at 500 °C for 5 h in an ambient environment to remove residual organics. A hydrostatic press (Daehatech, HP-1B) was used to press the powder into pellets, which were heated in a tubular furnace at 850 °C for 12 h at a heating rate of 5 °C/min under oxygen flow. Instead of slow cooling, the sample was quenched and preserved inside an inert gas-filled glove box. We prepared the P2-Na_2/3_Fe_1/2_Mn_1/2_O_2_ (NFM) material by following a similar approach, excluding the addition of magnesium nitrate salt. For details on structural, electrochemical, in situ, and ex situ characterizations, please see Supplementary Materials.

## Figures and Tables

**Table 1:** Crystallographic parameters for P2-Na_2/3_Mg_2/9_Fe_2/9_Mn_5/9_O_2_ compound refined by the Rietveld method.

Crystal System			Hexagonal		
Space Group			*P*6_3_/*mmc*		
Lattice Parameter			*a = b* = 2.91062 Å, c = 11.19962 Å α = 90°, β = 90°, γ = 120°		
Atom	*x*	*y*	*z*	Occupancy	B_iso_ (Å^3^ × 10^2^)
Na1	0.00000	0.00000	0.25000	0.119	6.380
Na2	0.33333	0.66667	0.75000	0.216	6.380
Fe	0.00000	0.00000	0.00000	0.110	1.215
Mn	0.00000	0.00000	0.00000	0.280	1.215
Mg	0.00000	0.00000	0.00000	0.110	1.215
O	0.33333	0.66667	0.08443	1.000	2.582
R_p_: 11.0, R_wp_: 15.4, R_exp_: 7.62, *χ^2^* = 4.10					

**Table 2:** ICP-OES results of the sample.

Theoretical Chemical Formula	Measured Atomic Ratio			
	Na	Mg	Fe	Mn
Na_0.67_Mg_0.22_Fe_0.22_Mn_0.56_O_2_	0.684	0.228	0.220	0.570

## Data Availability

All data and materials are available upon request from the corresponding author. The data are not publicly available due to ongoing research using a part of the data.

## Supplementary Materials

The following supporting information can be downloaded at: https://www.mdpi.com/article/10.3390/gels10010024/s1, Experimental details, Table S1: SEM-EDS analysis result for the P2-Na_2/3_Mg_2/9_Fe_2/9_Mn_5/9_O_2_ cathode material. Figure S1: High-resolution powder XRD pattern for the P2-Na_2/3_Mg_2/9_Fe_2/9_Mn_5/9_O_2_ (NFM-Mg) compound. Figure S2: (a) Galvanostatic voltage profiles, and (b) dQ/dV curves of the P2-Na_2/3_Fe_1/2_Mn_1/2_O_2_ (NFM) cathode material.
