# Supplementary material for: Electrochemical Storage Behavior of a High-Capacity Mg-Doped P2-Type Na2/3Fe1−yMnyO2 Cathode Material Synthesized by a Sol–Gel Method
Source: Gels. 2023 Dec 27;10(1):24. doi: 10.3390/gels10010024 (PMC10815866; doi:10.3390/gels10010024)
Supplement: Supplementary file 1 [file gels-10-00024-s001.zip › gels-2774049-supplementary.pdf]

Supporting Information

Electrochemical Storage Behavior of a High-Capacity Mg-Doped P2-Type $Na_{2/3}Fe_{1-y}Mn_yO_2$ Cathode Material Synthesized by a Sol–Gel Method

Mobinul Islam [1,*], Md. Shahriar Ahmed [1], Daseul Han [1], Gazi A. K. M. Rafiqul Bari [2] and Kyung-Wan Nam [1,*]

1 Department of Energy & Materials Engineering, Dongguk University, Seoul 04620, Republic of Korea; shahriar.che.sust@gmail.com (M.S.A.); endend42@naver.com (D.H.)
2 School of Mechanical Smart and Industrial Engineering, Gachon University, Seongnam 13120, Republic of Korea; grafiqulbari@gachon.ac.kr
* Correspondence: mobin85@dongguk.edu (M.I.); knam@dongguk.edu (K.-W.N.); Tel.: +82-2-2260-4978 (K.-W.N.)

Citation: Islam, M.; Ahmed, M.S.; Han, D.; Bari, G.A.K.M.R.; Nam, K.-W. Electrochemical Storage Behavior of a High-Capacity Mg-Doped P2-Type $Na_{2/3}Fe_{1-y}Mn_yO_2$ Cathode Material Synthesized by a Sol–Gel Method. *Gels* **2024**, *10*, 24. https://doi.org/10.3390/10.3390/gels10010024

Academic Editor: Viorel-Puiu Paun

Received: 30 November 2023
Revised: 23 December 2023
Accepted: 25 December 2023
Published: 27 December 2023

Copyright: © 2023 by the authors. Licensee MDPI, Basel, Switzerland. This article is an open access article distributed under the terms and conditions of the Creative Commons Attribution (CC BY) license (https://creativecommons.org/licenses/by/4.0/).

Experimental Characterizations

Structural Characterization

The structure of the as-synthesized P2-$Na_{2/3}Mg_{2/9}Fe_{2/9}Mn_{5/9}O_2$ material was studied using X-ray diffraction (Lab XRD, Rigaku X-ray diffractometer) technique. High-resolution Powder X-ray diffraction (HRPD) data were collected at 9B beamline of Pohang Acceleration Laboratory (PAL, South Korea) using a monochromated x-ray with a wavelength (λ) of 1.5183 Å.

Field emission-scanning electron microscopy (FE-SEM, JEOL-JSM-6700F) coupled with Energy Dispersive Spectroscopy (EDS) was used to examine the morphology and elemental mapping. The chemical composition and surface electronic states were investigated by X-ray photoelectron spectrometry (XPS, NEXSA, Thermo Fisher Scientific, USA). ICP-OES was performed using a Perkin Elmer 3000 DV optical emission plasma spectrometer.

Electrochemical Characterization

We made the slurry containing the synthesized active material, carbon black (Super P) and polyvinylidene fluoride (PVDF) binder (Sigma-Aldrich) with the weight ratio of 8:1:1 in N-methyl-2-pyrrolidone (NMP). The mixed slurry was cast onto an Al foil current collector. The prepared electrode was dried in a vacuum oven at 80 °C for 12 h prior to use as the working electrode. Then, coin-type half cells (CR2032) were assembled with a Na counter electrode, the prepared working electrode, a glass separator, and an electrolyte composed of 1.2 M $NaPF_6$ in ethylene carbonate/dimethyl carbonate (DMC)/ propylene carbonate (PC). (9:9:2 volume ratio). A battery testing system NEWARE (BT7.6.0) was used for the electrochemical test.

Hard and Soft X-ray Experiments

For ex-situ XRD and XAS analysis, the cells were disassembled in an argon-filled glove box, then the electrodes were carefully washed with propylene carbonate and dimethyl carbonate, and then vacuum sealed in a plastic bag after drying to protect them from exposure to air during transfer to the XRD and XAS chambers. The ex-situ XAS measurements of the Mn and Fe K-edge were performed at 7D, 8C and 10C beamlines of the Pohang Accelerations Laboratories (PAL). The energy was selected using a Si(111) double-crystal monochromator (detuned to ~40% of the maximum intensity). Pure cobalt and nickel metallic foils were used as a reference to cali-brate the respective spectra. The energy was scanned from -200 eV below to +1000 eV above the respective edges, and the spectra of the electrodes were recorded in the trans-mission mode. The XAS data were handled and processed using the ATHENA package. For soft X-ray (sXAS) experiments,

samples were transferred to the analysis chamber for testing under ultra-high vacuum. The ex-situ sXAS measurements of the Mn $L_{2,3}$-edge and O K-edge were performed at **10D and 10A1** beamlines of the Pohang Accelerations Laboratories (PAL). The soft X-ray absorption spectra (sXAS) in TFY and TEY mode were normalized by a gold mesh in the end station.

In-situ XRD

In situ XRD data were collected at **6D**-UNIST beamline of PAL in a transmission mode using X-ray with λ = 0.5949 Å. CR2032 coin cell with a 3 mm hole in the disk/lower/upper caps to allow the penetration of X-rays was used to conduct in situ XRD and cycling was conducted using a 10 mA g^{-1} current density.

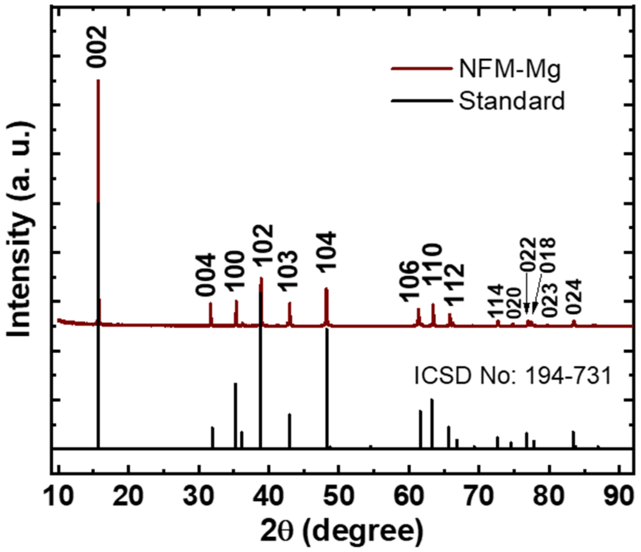

Figure S1. High-resolution powder XRD pattern for the P2-$Na_{2/3}Mg_{2/9}Fe_{2/9}Mn_{5/9}O_2$ (NFM-Mg) compound.

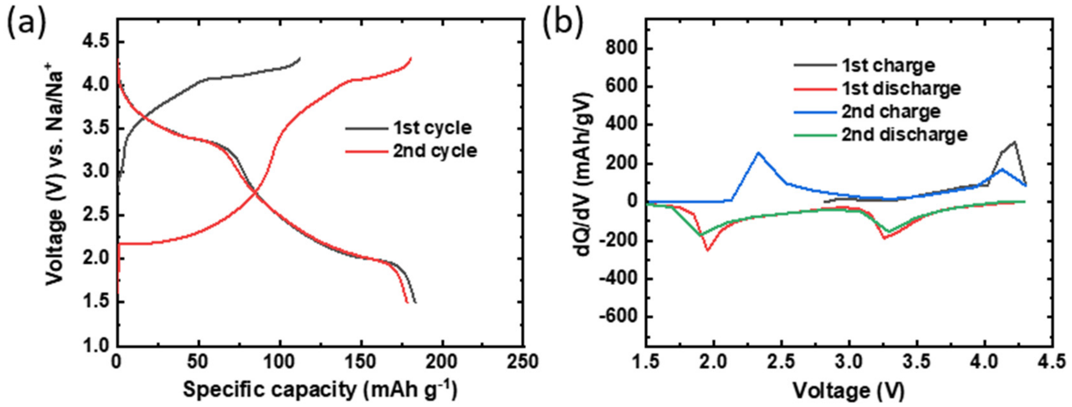

Figure S2. (a) Galvanostatic voltage profiles, and (b) dQ/dV curves of the P2-$Na_{2/3}Fe_{1/2}Mn_{1/2}O_2$ (NFM) cathode material.

Table S1. SEM-EDS analysis result for the P2-$Na_{2/3}Mg_{2/9}Fe_{2/9}Mn_{5/9}O_2$ cathode material.

Element	Weight%	MDL	Atomic%	Error%
O K	36.0	0.27	56.3	7.8
Na K	18.3	0.4	19.9	10.3
Mg K	5.2	0.3	5.4	11.1
Mn K	29.0	0.93	13.2	5.0
Fe K	11.5	1.39	5.1	8.9
